# Estudo comparativo da termoablação da veia safena magna na coxa, com e sem tumescência

**DOI:** 10.1590/1677-5449.004616

**Published:** 2016

**Authors:** Fabiano Luiz Erzinger, Walter Junior Boim de Araujo, Carlos Seme Nejm, Filipe Carlos Caron, Jorge Rufino Ribas Timi

**Affiliations:** 1 Instituto da Circulação, Serviço de Cirurgia Vascular, Curitiba, PR, Brasil.; 2 Universidade Federal do Paraná – UFPR, Departamento de Cirurgia, Curitiba, PR, Brasil.

**Keywords:** varizes, técnicas de ablação, terapia a laser

## Abstract

**Contexto:**

O tratamento com laser endovenoso das veias safenas oferece ao paciente um procedimento com baixos índices de complicações, proporcionando retorno precoce à atividade ocupacional.

**Objetivo:**

Comparar a formação de hematoma, a presença de parestesia no trajeto da veia safena magna (VSM) e a sua taxa de obliteração em 30 dias após a termoablação ao nível da coxa, utilizando ou não a tumescência e dois tipos de fibras.

**Métodos:**

Estudo prospectivo em que foram analisados três grupos de pacientes submetidos a termoablação da VSM em coxa, utilizando comprimento de onda 1470 nm. No grupo 1, utilizou-se fibra convencional e tumescência; no grupo 2, fibra convencional sem tumescência; e no grupo 3, fibra dupla radial sem tumescência. Foram comparados, no período de 30 dias, a taxa de obliteração ao eco-Doppler, a ocorrência de parestesias e hematomas.

**Resultados:**

Ao se comparar 90 VSMs de coxa submetidas a termoablação, obteve-se taxas de obliterações similares entre os grupos, sem diferença estatística. Nos grupos sem tumescência, ocorreu maior número de parestesias no trajeto da VSM na coxa no sétimo dia do que no grupo com tumescência, mas somente com significância estatística na comparação com o grupo da fibra convencional. Ocorreram hematomas em todos os grupos, sendo mais frequentes no grupo 1 (73,33%).

**Conclusões:**

A realização da tumescência mostrou-se útil na prevenção de lesões neurológicas menores, mas não influenciou a ocorrência de hematomas e a taxa de oclusão da VSM na coxa em até 30 dias de sua termoablação.

## INTRODUÇÃO

O tratamento cirúrgico das varizes com ligadura e extração da veia safena magna (VSM) e/ou parva, combinada com a excisão das varizes e ligadura das veias perfurantes insuficientes, tem sido o padrão de tratamento das varizes por mais de um século. Diversas adaptações técnicas foram realizadas com o passar do tempo: invaginação na fleboextração^1^^,^ 2, retirada de somente a safena da coxa para evitar lesão neurológica^3^, técnicas sem retirada da safena (Cure Conservatrice et Hemodynamique de l'Insufficience Veineuse en Ambulatoire – CHIVA)^4^.

O avanço na medicina está relacionado ao avanço tecnológico, buscando promover tratamentos menos invasivos e mais eficazes. Diversos equipamentos estão sendo desenvolvidos para melhorar os tratamentos existentes, como o uso de cateter para a realização de técnicas não ablativas, ocasionando lesão química e mecânica da veia safena simultaneamente^5^, assim como a utilização de cateter para liberação de cola (cianocrilato) na mesma^6^. Há também cateteres para a realização de técnicas termoablativas endovenosas, como a radiofrequência^7^ e o *laser*.

O tratamento com *laser* endovenoso das veias safenas iniciou-se nos anos 90. No entanto, em 2001, quando Navarro et al.^8^ publicaram seu primeiro artigo relevante sobre o tratamento da VSM com *laser* endovenoso, a técnica ganhou a atenção de toda a comunidade da flebologia. Hoje, o tratamento com *laser* endovenoso das veias safenas oferece ao paciente um procedimento que pode ser ambulatorial, permitindo retorno precoce à atividade ocupacional, na maioria dos casos. Também está associado a baixos índices de complicações, como hematomas e equimoses, contribuindo assim para um melhor resultado estético.

Nos últimos anos, fatores como a compreensão sobre o mecanismo de ação, o papel de vários comprimentos de onda do *laser*, os tipos de fibras, a necessidade ou não de tumescência e a energia adequada do *laser* estão gerando assunto para muitos estudos na área, que buscam verificar a eficácia e as complicações desse tratamento na comparação com o tratamento convencional^9^^-^ 13.

O objetivo deste estudo foi comparar a formação de hematoma, a presença de parestesia no trajeto da VSM e a taxa de obliteração em 30 dias após a termoablação ao nível da coxa, utilizando ou não a tumescência e dois tipos de fibras.

## MÉTODOS

**Figura 1:**
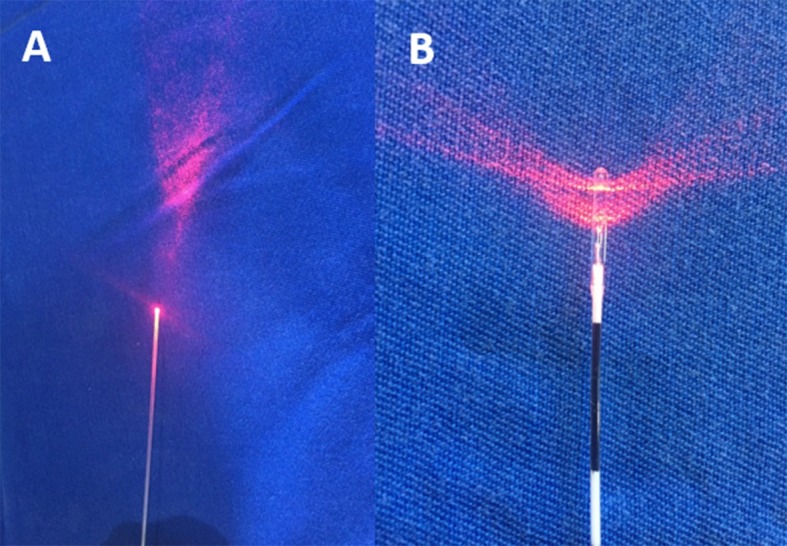
(A) fibra convencional; (B) fibra dupla radial.

Estudo prospectivo aprovado pelo Comitê de Ética em Pesquisa em Seres Humanos do Hospital de Clínicas da Universidade Federal do Paraná (HC-UFPR) (CAAE: 07643012.2.0000.0096), de acordo com as atribuições definidas na Resolução CNS 196/96. Foram analisados três grupos de pacientes submetidos a termoablação da VFM em coxa, sendo que no grupo 1 utilizou-se fibra convencional (*bare fiber*) e tumescência, no grupo 2 utilizou-se fibra convencional (*bare fiber*) sem tumescência e no grupo 3 utilizou-se fibra dupla radial sem tumescência (Figura 1). Foram avaliados a taxa de obliteração ao eco-Doppler e a ocorrência de parestesia e hematoma no período de 30 dias. Os grupos foram formados conforme a sequência de aparecimento dos pacientes para cirurgia, sendo os primeiros pacientes alocados ao grupo 1 e os últimos pacientes alocados ao grupo 3.

Os critérios de inclusão foram: pacientes maiores de 18 anos de ambos os sexos, com diagnóstico de varizes unilaterais ou bilaterais nos membros inferiores e indicação de tratamento cirúrgico com necessidade de tratamento da safena em coxa somente, associado ou não a flebectomias e/ou ao tratamento de veias perfurantes concomitantes, pertencentes às classes C2 a C6 da classificação *Clinical-Etiology-Anatomy-Physiopathology* (CEAP), e que concordaram em participar do estudo assinando o termo de consentimento livre e esclarecido, o qual foi devidamente explicado previamente.

Os critérios de exclusão foram: portadores de doença arterial periférica, pacientes com história prévia de trombose venosa profunda, pacientes com distúrbios hematológicos ou neurológicos dos membros inferiores, pacientes que estavam em uso de anticoagulantes e pacientes gestantes ou em amamentação. Nos grupos 2 e 3, além desses critérios, foram também excluídos os pacientes com safena magna fora do compartimento safênico ou então com a mesma estando visível ou palpável no exame físico.

Foram estudadas 30 VSMs em cada grupo, submetidas a termoablação na coxa utilizando fibra convencional (*bare fiber*) em dois grupos e fibra dupla radial em um grupo, todas com diâmetro de 600 µm, comprimento de onda 1470 nm e potência de 6 ou 7 W. A anestesia realizada foi raquianestesia ou peridural, a critério do anestesista.

**Figura 2:**
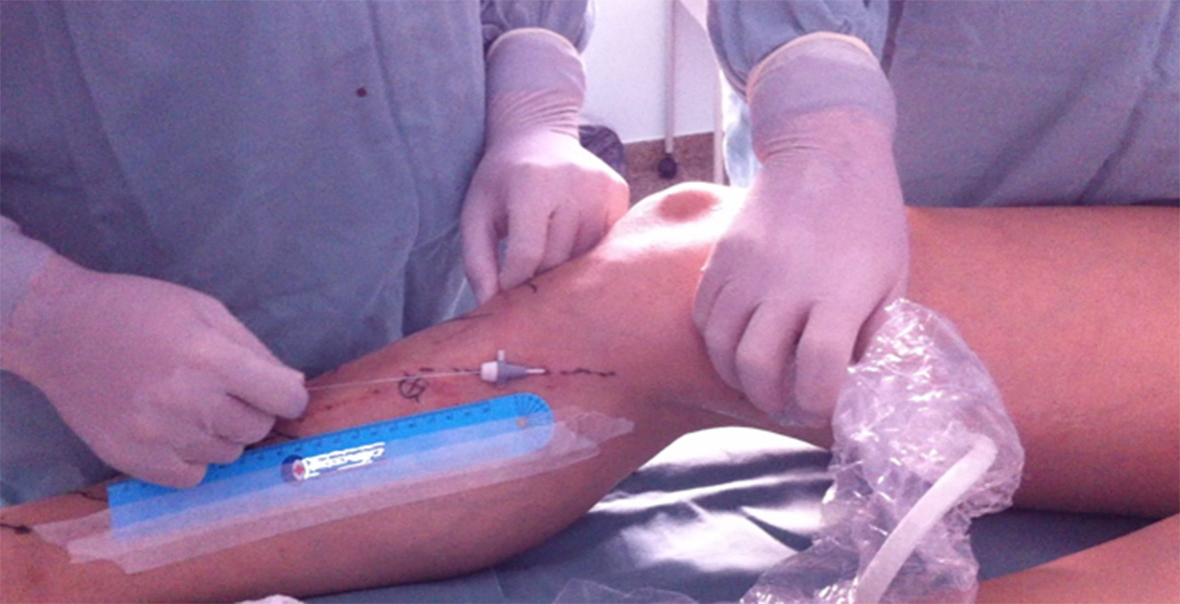
Local da punção e acompanhamento da progressão da fibra com ultrassom.

A fibra óptica foi introduzida na porção compreendida entre a porção distal da coxa e proximal da perna a ser tratada. Foi conduzida, guiada por ultrassom, no sentido anterógrado (Figura 2) até atingir a região inguinal, e a sua extremidade foi posicionada a cerca de 2 a 3 cm da junção safeno-femoral. Nos pacientes em que se optou por tumescência, foi realizada infiltração com soro fisiológico 0,9%, em temperatura ambiente, sendo ecoguiada no espaço da veia safena em todo o seguimento a ser tratado, até que a mesma ficasse colabada. Nos pacientes dos outros grupos, somente foi realizada a compressão manual ou com transdutor no trajeto da veia safena durante e após a termoablação, por 3 a 5 minutos. A fibra condutora do *laser* foi aos poucos tracionada manualmente, sem nenhum dispositivo mecânico, no sentido caudal até o local de término do tratamento desejado na coxa, com o paciente em posição de Trendelenburg. No pós-operatório, foram utilizados anti-inflamatórios e analgésicos comuns por 5 dias. Após a retirada das faixas, entre o terceiro e o quinto dia de pós-operatório, os pacientes passaram a fazer uso de meia elástica de média compressão (20-30 mmHg), meia-calça ou 7/8, evitando exercícios físicos por 15 dias. Os pacientes foram reexaminados através de exame físico para avaliação de parestesia e hematoma na coxa tratada, e de exame de eco-Doppler entre o quinto e o sétimo dia e no 30º dia para avaliação da taxa de obliteração da veia safena, incluindo também o estudo do sistema venoso profundo para excluir trombose venosa. O exame de eco-Doppler foi realizado por examinador independente, sem conhecimento do tipo de tratamento prévio, e o paciente foi mantido com o membro examinado em posição ortostática. Fluxo normal foi definido como anterógrado, e refluxo foi definido como fluxo retrógrado de 0,5 segundos de duração após uma manobra de Valsalva ou compressão manual e descompressão da parte distal do membro. A obliteração foi definida pela ausência de fluxo no segmento estudado.

O hematoma foi graduado como “menor” quando tinha até 25% do diâmetro da coxa e “maior” quando era maior que 25%, conforme exame visual direto (Tabela 1). A parestesia foi investigada clinicamente, com perguntas feitas ao paciente e também pela palpação direta de toda a coxa no trajeto da VSM, sendo caracterizada como presente ou ausente.

**Tabela 1:** Classificação da severidade do hematoma na coxa.

**Critério de severidade do hematoma**	**Área tratada com equimose (%)**
0	Nenhum
1	< 25
2	25-50
3	50-75
4	75-100
5	Extensão acima e abaixo do segmento tratado.

Para avaliação estatística e análise dos resultados do estudo, os números encontrados foram descritos por frequências e percentuais (variáveis qualitativas) ou por médias, medianas, valores mínimos, valores máximos e desvios padrões (variáveis quantitativas). Para a comparação dos grupos com relação às variáveis qualitativas, foi considerado o teste exato de Fisher ou o teste de qui-quadrado. Valores de p < 0,05 indicaram significância estatística. Os dados foram analisados com o programa computacional IBM SPSS Statistics v.20.

## RESULTADOS

Foram estudados 60 pacientes por 30 dias, totalizando 90 VSMs de coxa, que foram tratadas por termoablação com aparelho de 1470 nm e densidade de energia linear endovenosa (LEED) média de 33-53 J/cm. O diâmetro ao nível da junção safeno-femoral foi similar (média de 8 mm) nos três grupos, o mesmo acontecendo ao nível de coxa e joelho. Quanto à classificação da insuficiência venosa, mais da metade dos pacientes tinham CEAP C3. Foi necessária dissecção para acesso à veia safena interna somente em um paciente do grupo com tumescência; nos demais, o acesso foi por punção guiada por ultrassom na perna proximal ou na coxa distal.

As taxas de oclusão da VSM na coxa foram similares entre os grupos estudados no sétimo e no 30º dia. Apenas uma safena magna do grupo sem tumescência com fibra convencional não ocluiu no sétimo dia, apresentando redução do diâmetro inicial, sem refluxo. Com 30 dias de evolução, nos pacientes que foram submetidos a termoablação utilizando fibra convencional sem tumescência, observou-se que duas safenas nesse grupo evoluíram com refluxo segmentar em coxa (assintomático) e com diâmetros reduzidos. Não houve diferença estatística significativa entre os demais grupos, que apresentaram 100% de oclusão das safenas.

No grupo sem tumescência, ocorreu parestesia no trajeto da VSM na coxa no sétimo dia em número maior do que no grupo com tumescência (Tabela 2), mas somente com significância estatística quando comparada ao grupo da fibra convencional. Apesar de ser utilizada uma potência LEED maior no grupo da fibra dupla radial (35 e 33 J/cm *versus* 55 J/cm), a intensidade do desconforto foi leve em todos os grupos, sem necessidade de medicamento específico em nenhum deles. No controle de 30 dias, todos os grupos apresentaram porcentagens semelhantes de parestesia no trajeto da VSM na coxa (13,33% *versus* 23,33% *versus* 20,69%), ocorrendo melhora espontânea sem tratamento específico nesse período. Também não houve interferência no início das atividades habituais (Tabela 2).

**Tabela 2:** Comparação de hematomas, taxas de oclusão e parestesias.

	**Com tumescência e fibra convencional** **Grupo 1**	**p**	**Sem tumescência e com fibra convencional** **Grupo 2**	**Sem tumescência e com fibra dupla radial** **Grupo 3**
Hematoma menor em 7 dias	63,33%	0,003	20,0%	36,67%
Equimose presente em 30 dias	13,33%		3,85%	3,33%
Oclusão em 7 dias	100,0%		96,67%	100,0%
Oclusão em 30 dias	100,0%		93,33%	100,0%
Parestesia em 7 dias	23,33%	0,008	60,0%	50,0%
Parestesia em 30 dias	13,33%		23,33%	20,69%
LEED médio	35 J/cm		33 J/cm	55 J/cm

**Figura 3:**
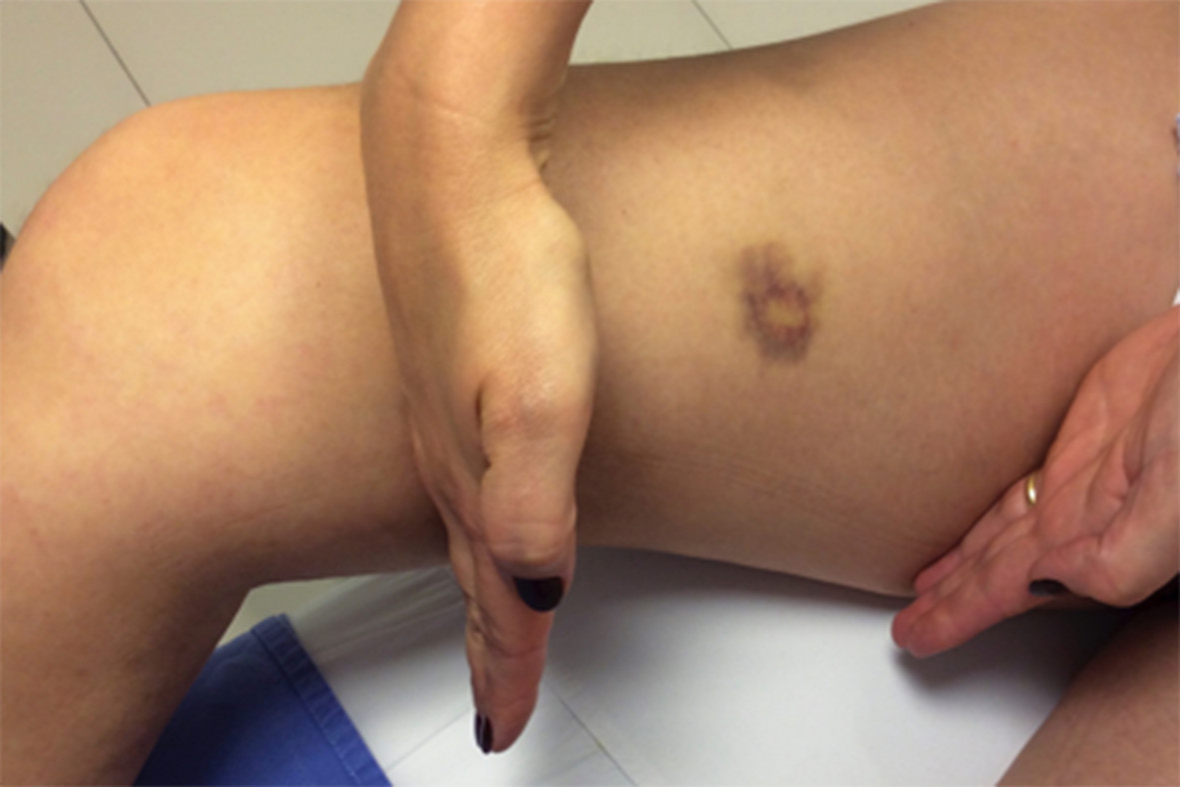
Controle no sétimo dia. Paciente do grupo com tumescência com hematoma menor, local da parestesia indicado.

Ocorreram hematomas em todos os grupos em 7 dias. Nos grupos sem tumescência, eles ocorreram em menos da metade dos pacientes, sendo mais frequentes os menores (até 25%). Já no grupo com tumescência, hematomas ocorreram em 73,33% dos pacientes (Figura 3), sendo os menores responsáveis por 63,33% dos casos (p = 0,003). Os hematomas maiores foram menos frequentes em todos os grupos nesse período. Os hematomas menores persistiram com maior frequência no grupo com tumescência, porém sem significância estatística, ao longo dos 30 dias subsequentes. Não ocorreram hematomas maiores nesse período, assim como não houve lesões térmicas na pele em nenhum momento (Tabela 3).

**Tabela 3:** Comparação dos hematomas.

**Hematomas em 7 dias**	**Grupo**		
	**Tumescência**	**Sem tumescência**	**Sem tumescência e com fibra dupla radial**
0	8	19	15
	26,67%	63,33%	50,0%
25	19	6	11
	63,33%	20,0%	36,67%
50	3	4	4
	10,0%	13,33%	13,33%
75	0	1	0
	0,0%	3,33%	0,0%
Total	30	30	30

Não ocorreu nenhum caso de trombose venosa profunda, mas foram verificados dois casos de trombose endovenosa induzida pelo calor (EHIT) da veia safena, que se estenderam até a veia femoral. Em ambos os casos, a extensão do trombo era inferior a 5 cm e não ultrapassava 50% da luz, sendo tratados com anticoagulante e desaparecendo no controle de eco-Doppler de 30 dias. Um caso ocorreu no grupo de fibra convencional com tumescência e outro no grupo sem tumescência.

## DISCUSSÃO

Na maioria das vezes, as varizes ocorrem devido à incompetência da VSM com ou sem perfurantes incompetentes. O tratamento convencional para safena é a ligadura sobre a junção safeno-femoral e a remoção da mesma, o que geralmente implica anestesia geral ou raquidiana. Em muitos centros, os pacientes são submetidos a operação, sendo hospitalizados por 12 a 24 horas. A termoablação venosa com *laser* endovenoso foi introduzida como alternativa à ligadura e remoção da veia safena magna e parva por Navarro et al.^8^ em 2001. O mecanismo de ação da termoablação a *laser* (EVLT) é produzir calor, resultando em dano endotelial, trombose e fibrose da veia^10^.

Numerosos estudos mostram que o EVLT tem se tornado um tratamento eficaz para varizes dos membros inferiores, com baixas taxas de recorrência e poucas complicações relacionadas ao procedimento – tromboflebite (7%), queimadura na pele (< 1%), hiperpigmentação (5%), parestesias (1% a 2%), formação de hematomas (até 7%). No entanto, ainda não dispomos de resultados em longo prazo^13^.

A dor nos pacientes tratados com EVLT é menor quando comparada a de pacientes submetidos a safenectomia, assim como a formação de edema no pós-tratamento, ocorrendo assim uma recuperação mais rápida para o retorno às atividades laborais^11^^-^ 17. Isso é corroborado pelas diretrizes da Society for Vascular Surgery (SVS) e do Fórum Venoso Americano (FAV), que qualificam o tratamento por técnicas ablativas para varizes dos membros inferiores com grau de recomendação 1 e nível de evidência B. Já para a cirurgia convencional, o nível é 2b^18^^,^ 19.

No intuito de minimizar ainda mais as complicações e melhorar a eficácia do método no tratamento das varizes, têm-se estudado formas mais seguras e eficazes de se entregar a energia para o efeito termoablativo, seja na forma de comprimentos de onda, tipos de fibras, velocidade de tração da fibra, intensidade de energia e importância da tumescência.

A tumescência proporciona uma proteção aos tecidos perivasculares, principalmente os nervos e a pele, dos efeitos térmicos da energia intravascular, servindo como um dissipador de calor. Auxilia também a diminuir o diâmetro da veia tratada para permitir uma melhor absorção da energia pelo cromóforo-alvo, assegurando que as veias de grande diâmetro sejam comprimidas de forma adequada sob a orientação ecográfica^17^.

A energia da fibra com 1470 nm de comprimento de onda é absorvida, preferencialmente pela água, até 40 vezes mais do que o comprimento de onda de 980 nm, em que o cromóforo-alvo é a hemoglobina. Assim, aquela necessita de menor energia para ocasionar lesão na parede da veia, causando menor dor e menos equimoses quando comparada com as fibras de 980 nm^15^^,^ 16 ^,^ 20. A utilização de fibra convencional, pela forma de emissão unidirecional, tem uma tendência maior de perfuração da parede venosa e apresenta maior destruição das estruturas ao redor da veia. As fibras radial e dupla radial foram criadas com o intuito de aumentar a área de contato associada a uma forma homogênea de distribuição da energia na parede da veia com baixa penetração, o que poderia contribuir para menor intensidade da dor e menos hematomas, necessitando de um LEED menor para fazer o tratamento^13^^,^ 21.

Os hematomas nos primeiros 7 dias ocorreram de maneira mais frequente no grupo com tumescência (63,33% *versus* 20%), provavelmente devido a uma perfuração acidental da veia safena durante a realização da punção, mas que não contribuiu para prejuízo em 30 dias e não necessitou de tratamento adicional. A ocorrência de hematoma no grupo de fibra dupla radial provavelmente ocorreu devido a uma maior taxa de perfuração, que deve estar relacionada à maior energia empregada (LEED 55 *versus* 33 e 35), porém sem diferença estatística quando comparada com os demais grupos.

Neste estudo, verificou-se que a taxa de oclusão foi menor, porém sem diferença estatística nos pacientes submetidos a tratamento com fibra convencional e sem tumescência. Observou-se que a tumescência influenciou (de maneira não significativa) a taxa de oclusão, contribuindo para sua eficácia quando utilizadas fibra convencional e baixa energia (LEED), o que também ocorreu no estudo de Araujo et al.^22^, que verificaram uma recanalização maior em 6 meses utilizando tais parâmetros.

Pode-se verificar que a utilização de tumescência contribuiu para a taxa de oclusão e para uma proteção contra a frequência da parestesia quando associada ao uso de fibra convencional. No entanto, tal benefício não se tornou evidente ao se utilizar a fibra dupla radial, que mostrou grande eficácia em 30 dias, assim como baixos índices de complicações como parestesias e hematomas. A taxa de oclusão em 30 dias, com uso de baixa energia (LEED de 30 J/cm) e fibra convencional, mostrou-se eficaz em ambos os grupos, com possível influência da tumescência no resultado. Estudos com acompanhamento mais prolongado, com maior número de safenas, devem ser realizados para determinar a influência da tumescência como fator determinante na taxa de oclusão da safena na coxa, considerando que obtivemos duas safenas não ocluídas sem a realização da tumescência, mas com redução do seu calibre. Quando utilizamos a fibra dupla radial com LEED maior, foi obtida uma taxa de oclusão de 100%. Portanto, existem outros fatores a serem considerados que podem influenciar os resultados, como o tipo de fibra (radial ou dupla radial) e a utilização de maior energia (LEED), além da própria tumescência, a qual em outros estudos foi responsável por um maior número de complicações imediatas, sem melhorar a eficácia^23^^,^ 24.

Ao se utilizar a fibra dupla radial, não houve necessidade de tumescência para alcançar uma taxa de 100% de oclusão nos pacientes em 30 dias, assim como demonstrado no estudo de Galego et al.^25^ com fibra radial e sem tumescência. Mesmo utilizando menor energia (LEED de 30 J/cm), não houve comprometimento do resultado tardiamente. Pode-se pensar em utilizar tal quantidade de energia, já que, neste estudo, verificou-se uma taxa de hematomas um pouco maior, assim como na intenção de diminuir a chance de parestesia sem comprometer a taxa de oclusão.

A quantidade de energia (LEED) utilizada na termoablação é um fator que deve ser levado em conta, pois, acima de 100 J/cm, está relacionado a uma taxa de 7,6% de parestesia no trajeto da veia safena pós-tratamento^26^. Ao se utilizar 35, 60 ou 80 J/cm, é possível proporcionar o fechamento adequado da veia; portanto, utilizou-se um LEED seguro para o tratamento com fibra convencional e radial^15^^,^ 27 ^,^ 28.

Com relação à frequência da parestesia, verificou-se que a tumescência exerceu papel protetivo quanto à frequência da sua ocorrência no pós-operatório, sendo significativa (23,33% *versus* 60%) quando comparada nos primeiros 7 dias com a fibra convencional. A diferença não foi estatisticamente significativa no grupo da fibra dupla radial, mesmo utilizando maior energia. Ao longo de 30 dias, a melhora ocorreu nos grupos com e sem tumescência, mas uma melhora superior a 50% foi verificada nos grupos sem tumescência. Isso representa a ocorrência de uma lesão neurológica leve, porém mais frequente, o que demonstra que a proteção da tumescência não influenciou a intensidade da lesão neurológica, mas só a sua frequência. O efeito benéfico maior com a realização da tumescência está mais relacionado ao efeito da anestesia tumescente, cujo propósito é realizar um procedimento ainda menos invasivo. Assim, evita-se anestesia geral ou bloqueio regional, que favorecem a imobilidade, um fator de risco para a ocorrência da trombose venosa profunda. Essa anestesia diminui o risco do procedimento pela deambulação precoce e por não haver necessidade de internamento hospitalar^24^.

A realização da tumescência mostrou-se útil na prevenção de lesões neurológicas menores, mas não influenciou a prevenção de hematomas nem a taxa de oclusão da safena magna na coxa em até 30 dias da termoablação. A fibra dupla radial apresentou melhores resultados.
