# Supplementary material for: Estudo comparativo da termoablação da veia safena magna na coxa, com e sem tumescência
Source: J Vasc Bras. 2016 Jul-Sep;15(3):217–23. [Article in Portuguese] doi: 10.1590/1677-5449.004616 (PMC5829759; doi:10.1590/1677-5449.004616)
Supplement: Translation

# Translation: Comparative study of great saphenous vein ablation in the thigh, with and without tumescence

**Authors:** Fabiano Luiz Erzinger, Walter Junior Boim de Araujo, Carlos Seme Nejm, Filipe Carlos Caron, Jorge Rufino Ribas Timi

**Affiliations:** 1 Instituto da Circulação, Serviço de Cirurgia Vascular, Curitiba, PR, Brazil.; 2 Universidade Federal do Paraná – UFPR, Departamento de Cirurgia, Curitiba, PR, Brazil.

**Keywords:** varicose veins, ablation techniques, laser treatment

## Abstract

**Background:**

Endovenous laser treatment of saphenous veins offers patients a procedure with low rates of complications and an early return to occupational activities.

**Objective:**

To compare rates of formation of bruising, of paresthesia along the path of the great saphenous vein (GSV), and of GSV obliteration 30 days after thermal ablation in the thigh, performed with or without tumescence and using two different types of fiber.

**Methods:**

This was a prospective study, analyzing three groups of patients who underwent GSV thermal ablation in the thigh, using a wavelength of 1470 nm. Patients in group 1 were treated with a conventional fiber using tumescence; those in group 2 were treated with a conventional fiber without using tumescence; and patients in group 3 were treated with a double radial fiber without tumescence. After 30 days, the rates of obliteration shown by Doppler ultrasonography, of paresthesias, and of bruising were compared.

**Results:**

Comparison of the results of thermal ablation of 90 GSVs in the thigh revealed similar rates of obliteration, with no statistical differences. The rate of paresthesia along the path of the GSV in the thigh was higher in the groups without tumescence than in the group with tumescence, but the difference only attained statistical significance for the comparison with the group that was treated with the conventional fiber. There was bruising in all groups, with greater frequency in group 1 (73.33%).

**Conclusions:**

Tumescence proved useful for preventing minor neurological injuries, but didn’t have any influence on the rates of bruising occurrence or of occlusion of the GSV in the thigh up to 30 days after thermal ablation.

## INTRODUCTION

For more than a century, surgical treatment with ligature and stripping of the great saphenous vein (GSV) and/or the small saphenous vein, combined with excision of varicose veins and ligature of incompetent perforating veins was the standard treatment for varicose veins. Several technical adaptations were introduced over the years: invagination stripping,^1^^,^ 2 removal of only the segment of saphenous vein in the thigh to avoid neurological injuries,^3^ and techniques that do not involve removal of the saphenous vein (Cure Conservatrice et Hemodynamique de l'Insufficience Veineuse en Ambulatoire – CHIVA).^4^

Advances in medicine are related to technological developments designed to provide less invasive and more effective treatments. Many different types of equipment and techniques are under development to improve existing treatments, such as catheters for non-ablative techniques in which the saphenous vein undergoes chemical and mechanical damage simultaneously,^5^ or use of catheters to release glue (cyanoacrylate) into the vein.^6^ There are also catheters designed for endovenous thermoablation techniques, with radio frequency^7^ or laser.

Treatment of the saphenous veins with endovenous laser began in the 1990s. However, it was in 2001, when Navarro et al.^8^ published their first well-received article on treatment of the GSV with endovenous laser that the technique caught the attention of the entire phlebology community. Nowadays, treatment of the saphenous veins with endovenous laser offers patients a procedure that can be performed in ambulatory settings and allows them an early return to occupational activities, in the majority of cases. It is also associated with low rates of complications, such as bruising and ecchymosis, further contributing to better esthetic results.

Over recent years, attempts to understand factors such as the mechanism of action, the role of different laser wavelengths and fiber types, whether or not tumescence is needed, and the ideal laser energy to employ are the underlying objectives of many studies in this area, designed to determine the efficacy and complications of these treatments in comparison with conventional treatment.^9^^-^ 13

The objective of this study was to compare formation of bruising, occurrence of paresthesia along the path of the GSV, and the rate of obliteration 30 days after thermal ablation was conducted at the level of thigh, with or without tumescence and with one of two different types of fiber.

## METHODS

This was a prospective study, approved by the Human Research Ethics Committee at the Hospital de Clínicas da Universidade Federal do Paraná (HC-UFPR) (Ethics approval certificate: 07643012.2.0000.0096) and conducted as mandated by the relevant legislation. An analysis was conducted of three groups of patients who underwent thermal ablation of the GSV in the thigh. Group 1 patients were treated with a conventional bare fiber and tumescence, those in group 2 were treated with a conventional bare fiber, but without tumescence, and group 3 patients were treated using a double radial fiber without tumescence (Figure 1). Doppler ultrasonography was used to determine the rate of obliteration, which was compared between groups together with rates of paresthesia and bruising over a 30-day period. Patients were allocated to groups sequentially as they were referred for surgery, with those attending first assigned to group 1 and the last to group 3.

**Figure 1:** (A) Conventional (bare) fiber; (B) Double radial fiber.

Inclusion criteria were as follows: patients of both sexes; over the age of 18; with a diagnosis of unilateral or bilateral varicose veins in the lower limbs; referred for surgical treatment; requiring treatment of the saphenous vein in the thigh only, with or without concurrent phlebectomies and/or treatment of perforating veins; classified as categories C2 to C6 according to the Clinical-Etiology-Anatomy-Physiopathology (CEAP) system; and who agreed to take part in the study, signing a free and informed consent form, which had been duly explained to them in advance.

The exclusion criteria were as follows: peripheral arterial disease; patients with a prior history of deep venous thrombosis; patients with hematological or neurological disorders of the lower limbs; patients taking anticoagulants; and pregnant or breastfeeding women. In groups 2 and 3, in addition to application of these criteria, patients were also excluded if their great saphenous veins were not within the saphenous compartment or were visible or palpable during the physical examination.

A total of 30 GSVs were studied in each group after thermal ablation in the thigh using a conventional bare fiber in two groups and a double radial fiber in one group, all with diameter of 600 µm, wavelength of 1470 nm, and power of 6 or 7 W. Spinal or epidural anesthesia was conducted at the anesthetist’s discretion.

The optical fiber was introduced into the segment extending from the distal to the proximal portion of the thigh of the leg to be treated. It was advanced in the anterograde direction, guided with ultrasound (Figure 2), until it reached the groin and the tip was positioned around 2 to 3 cm from the saphenofemoral junction. For patients allocated to receive tumescence (group 1), 0.9% saline solution was infiltrated at room temperature, guided into the space around the saphenous vein with ultrasound all along the segment to be treated, until the vein became compressed. For patients in the other two groups, compression was applied manually or with the transducer along the path of the saphenous vein during and after thermal ablation, for 3 to 5 minutes. The laser fiber was gradually tractioned manually, without using a mechanical device, in the distal direction until the end of the planned treatment area of the thigh, with the patient in the Trendelenburg position. During the postoperative period, standard anti-inflammatories and analgesics were prescribed for 5 days. After removal of bandages, between 3 and 5 days after the procedure, patients began to wear medium compression (20-30 mmHg), elastic hosiery, avoiding physical exercise for 15 days. Patients attended another physical examination for assessment of paresthesia and bruising in treated thighs, and Doppler ultrasonography was conducted at between 5 and 7 days and once more at 30 days after the procedure, to determine the rate of saphenous vein obliteration and to study the deep vein system to rule out venous thrombosis. Doppler ultrasonography was conducted by an independent examiner, who was blind to the type of treatment provided, and patients were examined with the treated limb in orthostatic position. Normal flow was defined as anterograde, and reflux was defined as retrograde flow with a duration of 0.5 seconds after a Valsalva maneuver or manual compression and decompression of the distal portion of the limb. Obliteration was defined as an absence of flow in the segment being studied.

**Figure 2:** Puncture site and monitoring of progress of fiber with ultrasound.

Bruising was graded as “minor” when it involved up to 25% of the diameter of the thigh and “major” when it involved more than 25%, by direct visual observation (Table 1). Paresthesia was investigated clinically, by questioning the patient and by direct palpation of the whole thigh along the path of the GSV, and was classified as present or absent.

**Table 1:** Classification of the severity of bruising in the thigh.

**Severity of bruising criterion**	**Percentage of treated area with ecchymosis**
0	Zero
1	< 25
2	25-50
3	50-75
4	75-100
5	Extends above and below the segment treated.

For statistical assessment and analysis of the study results, the numbers recorded were described in terms of frequencies and percentages (qualitative variables) or means, medians, ranges, and standard deviations (quantitative variables). Qualitative variables were compared between groups using Fisher’s exact test or the chi-square test. P values p < 0.05 were considered indicative of statistical significance. Data were analyzed with IBM SPSS Statistics v.20.

## RESULTS

A total of 60 patients and 90 GSVs treated in the thigh were studied for 30 days. These patients were treated by thermoablation with using a wavelength of 1470 nm and a mean linear intravenous energy density (LEED) of 33-53 J/cm. Vein diameters at the level of the saphenofemoral junction were similar in all three groups (mean of 8 mm) and the same was true at the thigh and knee. More than half of the patients had a venous insufficiency classification of CEAP C3. Dissection was only needed to access the internal saphenous vein in one patient in the group with tumescence; in all other patients, access was achieved by ultrasound-guided puncture of the proximal leg or distal thigh.

Rates of occlusion of the GSV in the thigh were similar in the three study groups at 7 and at 30 days. Just one great saphenous vein had not occluded by day 7, in the group treated without tumescence using the conventional fiber, exhibiting a reduction compared to the initial diameter, without reflux. However, at 30 days after the procedure, two saphenous veins had developed (asymptomatic) reflux in segments in the thigh, with reduced diameters, both in patients who had undergone thermal ablation with a bare fiber and without tumescence. There were no statistically significant differences between the other groups, both of which exhibited 100% occlusion of saphenous veins.

In the group treated without tumescence, there was a higher rate of paresthesia along the path of the GSV in the thigh on day 7 than in the group treated with tumescence (Table 2), but the difference only attained statistical significance for the comparison with the bare fiber group. Although a higher LEED was used in the group treated with the double radial fiber (35 and 33 J/cm vs. 55 J/cm), the intensity of discomfort was mild in all three groups and none of them needed administration of specific medication. At the 30-day follow-up consultations, all groups exhibited similar percentages of paresthesia along the path of the GSV in the thigh (13.33% vs. 23.33% vs. 20.69%), and recoveries were spontaneous with no specific treatment during the period. There was also no negative impact on normal daily activities (Table 2).

**Table 2:** Comparison of bruising, occlusion rates, and paresthesias.

	**With tumescence and conventional fiber** **Group 1**	**p**	**Without tumescence and with conventional fiber** **Group 2**	**Without tumescence and with double radial fiber** **Group 3**
Minor bruising at 7 days	63.33%	0.003	20.0%	36.67%
Ecchymosis present at 30 days	13.33%		3.85%	3.33%
Occlusion at 7 days	100.0%		96.67%	100.0%
Occlusion at 30 days	100.0%		93.33%	100.0%
Paresthesia at 7 days	23.33%	0.008	60.0%	50.0%
Paresthesia at 30 days	13.33%		23.33%	20.69%
Mean LEED	35 J/cm		33 J/cm	55 J/cm

There was bruising in all groups at 7 days. In groups without tumescence bruising occurred in less than half of the patients, and for those in which it did occur, minor bruising was more common. In contrast, in the group treated with tumescence, bruising occurred in 73.33% of the patients (Figure 3) and 63.33% of cases were classed as minor (p = 0.003). Major bruising was less frequent in all groups over 7 days. Minor bruising that persisted up to 30 days was more frequent in the group treated with tumescence, but without statistical significance. At this point no major bruising remained and there were no thermal skin injuries at any point (Table 3).

**Figure 3:** Control on day 7. Patient in group treated with tumescence, with minor bruising, indicating location of paresthesia.

**Table 3:** Comparison of bruising rates.

**Bruising at 7 days**	**Group**		
	**With tumescence**	**Without tumescence**	**Without tumescence and with double radial fiber**
0	8	19	15
	26.67%	63.33%	50.0%
25	19	6	11
	63.33%	20.0%	36.67%
50	3	4	4
	10.0%	13.33%	13.33%
75	0	1	0
	0.0%	3.33%	0.0%
Total	30	30	30

There were no cases of deep venous thrombosis, but there were two cases of endovenous heat-induced thrombosis (EHIT) in saphenous veins with extension up to the femoral vein. In both cases the thrombus had an extension of less than 5 cm and did not obstruct more than 50% of the lumen, so they were treated with anticoagulants and had disappeared by the 30-day control Doppler ultrasonography. One of these cases was in the bare fiber with tumescence group and the other was in the group treated without tumescence.

## DISCUSSION

In the majority of cases, varicose veins are the result of GSV incompetence, with or without incompetent perforating veins. Conventional treatment of the saphenous vein consists of ligature above the saphenofemoral junction and removal, which generally requires either general or spinal anesthesia. At many centers patients are still treated surgically and are hospitalized for 12 to 24 hours. Venous thermal ablation with endovenous laser was introduced by Navarro et al.^8^ in 2001 to provide an alternative to ligature and removal of the great and small saphenous veins. The mechanism of action of endovenous laser thermal ablation (EVLT) is production of heat, resulting in endothelial damage, thrombosis, and fibrosis of the vein.^10^

A large number of studies have shown that EVLT has become an effective treatment for varicose veins of the lower limbs, with low rates of recurrence and of complications related to the procedure – thrombophlebitis (7%), skin burns (< 1%), hyperpigmentation (5%), paresthesias (1% to 2%), and formation of bruising (up to 7%). However, we do not yet have long-term results.^13^

The pain suffered by patients treated with EVLT is less than that reported by patients treated with saphenectomy, and the same is true of formation of edema after treatment, with the result that recovery is faster, allowing an earlier return to work.^11^^-^ 17 This is confirmed by guidelines published by the Society for Vascular Surgery (SVS) and the American Venous Forum (AVF), which have classified treatment of varicose veins of the lower limbs with ablative techniques at a recommendation level of 1 with evidence level B. The equivalent for conventional surgery is 2b.^18^^,^ 19

Attempts to reduce the rates of complications and improve the efficacy of this method for treatment of varicose veins even further have focused on safer and more effective methods of delivering the energy used to achieve the thermoablative effect, studying wavelengths, types of fibers, fiber traction velocity fiber, energy intensity, and the importance of tumescence.

Tumescence provides protection for the perivascular tissues (primarily nerves and skin) from the thermal effects of the endovenous energy, working to dissipate the heat. It also helps to reduce the diameter of the vein being treated, to enable better energy absorption by the target chromophore, ensuring that large diameter veins are compressed sufficiently under ultrasound guidance.^17^

The energy emitted by the fiber at a wavelength of 1470 nm is preferentially absorbed by water, up to 40 times more than the 980 nm wavelength, for which the target chromophore is hemoglobin. The result is that at 1470 nm less energy is needed to damage the wall of the vein, causing less pain and less ecchymosis when compared with fibers that deliver energy at 980 nm.^15^^,^ 16 ^,^ 20 Additionally, using conventional bare fibers, with unidirectional emission results in a greater tendency to perforate the vein wall and a greater likelihood that structures around the vein will be destroyed. Radial and double radial fibers were developed with the intention of increasing the area of contact and achieving a more uniform distribution of energy at the vein wall, combined with a lower rate of penetration, which could result in lower intensity pain and less bruising, because a lower LEED would be needed to accomplish treatment.^13^^,^ 21

Bruising during the first 7 days was more frequent in the group treated with tumescence (63.33% vs. 20%), probably because of accidental perforation of the saphenous vein during puncture, but this did not increase the rate of complications at 30 days and did not require additional treatment. The bruising in the double radial fiber group was probably caused by a higher rate of perforation, which is probably related to the greater quantity of energy employed (LEED 55 J/cm vs. 33 and 35 J/cm), but there was no statistical difference when compared to the other groups.

In this study, it was found that there was a lower rate of occlusion among patients treated with the bare fiber and without tumescence, although the difference did not attain statistical significance. It was observed that tumescence had a (non-significant) influence on occlusion rate, contributing to efficacy when used with a conventional fiber and low energy (LEED), which was also observed in a study by Araujo et al.,^22^ who observed a greater recanalization rate at 6 months using these parameters.

It was found that tumescence contributed to the occlusion rate and to protection against the frequency of paresthesia when combined with the bare fiber. However, this benefit was not maintained in the comparison with the double radial fiber, which achieved high efficacy at 30 days combined with low rates of complications such as paresthesias and bruising. The occlusion rate at 30 days for the combination of low energy (LEED of 30 J/cm) and the conventional fiber demonstrated good efficacy in both groups, with a possible influence from tumescence on the result. Studies should be conducted with longer follow-up and samples containing larger numbers of saphenous veins in order to determine the influence of tumescence as a determinant factor in the rate of occlusion of saphenous veins in the thigh, since we had two saphenous veins that were not occluded after treatment without tumescence, although their caliber had been reduced. Using the double radial fiber with a higher LEED, we obtained a 100% occlusion rate. As such, there are other factors to be considered that could be affecting the results, such as the type of fiber (radial or double radial) and utilization of greater quantities of energy (LEED), in addition to tumescence, which in other studies was responsible for a larger number of immediate complications, without improving efficacy.^23^^,^ 24

When treating with the double radial fiber, it was not necessary to employ tumescence to achieve 100% occlusion at 30 days, as has been shown in a study published by Galego et al.^25^ with a radial fiber and no tumescence. Even using a lower quantity of energy (a LEED of 30 J/cm), there was no deterioration of results later on. It is worth considering using this lower level of energy, since in the present study we observed a slightly higher rate of bruising and also with a view to reducing the likelihood of paresthesia without compromising the occlusion rate.

The quantity of energy (LEED) used for thermal ablation is a factor that must be considered because rates above 100 J/cm, are associated with a 7.6% rate of paresthesia along the path of the saphenous vein after treatment.^26^ It is possible to provoke adequate closure of the vein using 35, 60 or 80 J/cm; so a safe LEED level was used for treatment with both the bare and the radial fibers.^15^^,^ 27 ^,^ 28

With relation to the frequency of paresthesia, it was found that tumescence played a protective role with regard to occurrence of paresthesia during the postoperative period, and the comparison with the conventional fiber at 7 days was significant (23.33% vs. 60%). The difference was not statistically significant for the comparison with the double radial fiber group, even though the latter was treated with a greater quantity of energy. Over the course of 30 days, patients in groups with and without tumescence improved, but improvements greater than 50% were observed in the groups without tumescence. This represents neurological injuries that were mild, but more frequent, showing that the protection afforded by tumescence did not influence the intensity of neurological injuries, just their frequency. The most beneficial effect of tumescence is linked to the effect of tumescent anesthesia, which is administered with the objective of making the procedure even less invasive. This avoids general anesthesia or regional blockades, which provoke immobility – a risk factor for deep venous thrombosis. This type of anesthesia reduces the risks of the procedure by allowing early mobilization and because it makes hospital admission unnecessary.^24^

Tumescence proved useful for prevention of minor neurological injuries, but did not have an effect in terms of prevention of bruising or on the rate of occlusion of the great saphenous vein in the thigh up to 30 days after thermal ablation. The double radial fiber produced the best results.
